# Supplementary material for: TiO2 Passivation Layer on ZnO Hollow Microspheres for Quantum Dots Sensitized Solar Cells with Improved Light Harvesting and Electron Collection
Source: Nanomaterials (Basel). 2020 Mar 28;10(4):631. doi: 10.3390/nano10040631 (PMC7221611; doi:10.3390/nano10040631)
Supplement: Supplementary file 1 [file nanomaterials-10-00631-s001.pdf]

Supplementary Materials

TiO_2 Passivation Layer on ZnO Hollow Microspheres for Quantum Dots Sensitized Solar Cells with Improved Light Harvesting and Electron Collection

Zhen Li [1,2,*], Libo Yu [1,2,*], Hao Wang [1], Huiwen Yang [1] and Huan Ma [1]

[1] College of Chemistry and Chemical Engineering, Hexi University, Zhangye City 734000, China; 2046871512@qq.com (H.W.); 2046871512@qq.com (H.Y.); 2046871512@qq.com (H.M.)

[2] Key Laboratory of Hexi Corridor Resources Utilization of Gansu, Hexi University, Zhangye City 734000, China

* Correspondence: lizhen@hux.edu.cn (Z.L.); yulibo@hxu.edu.cn (L.Y.)

Received: 7 March 2020; Accepted: 24 March 2020; Published: 28 March 2020

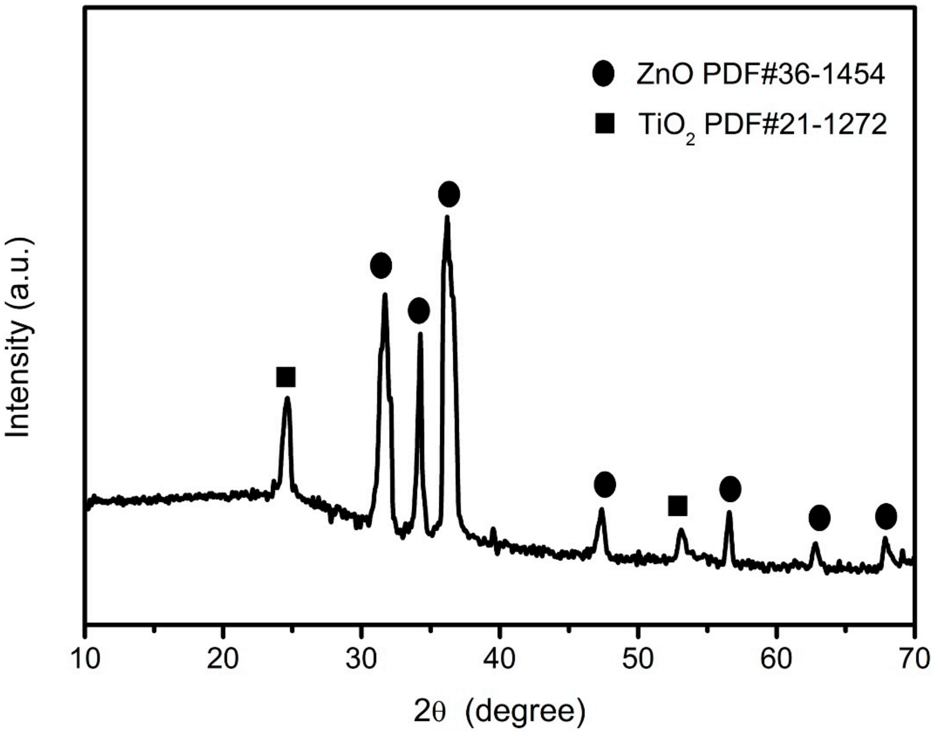

Figure S1. The XRD pattern of ZnO HMS passivated with TiO_2 layer.

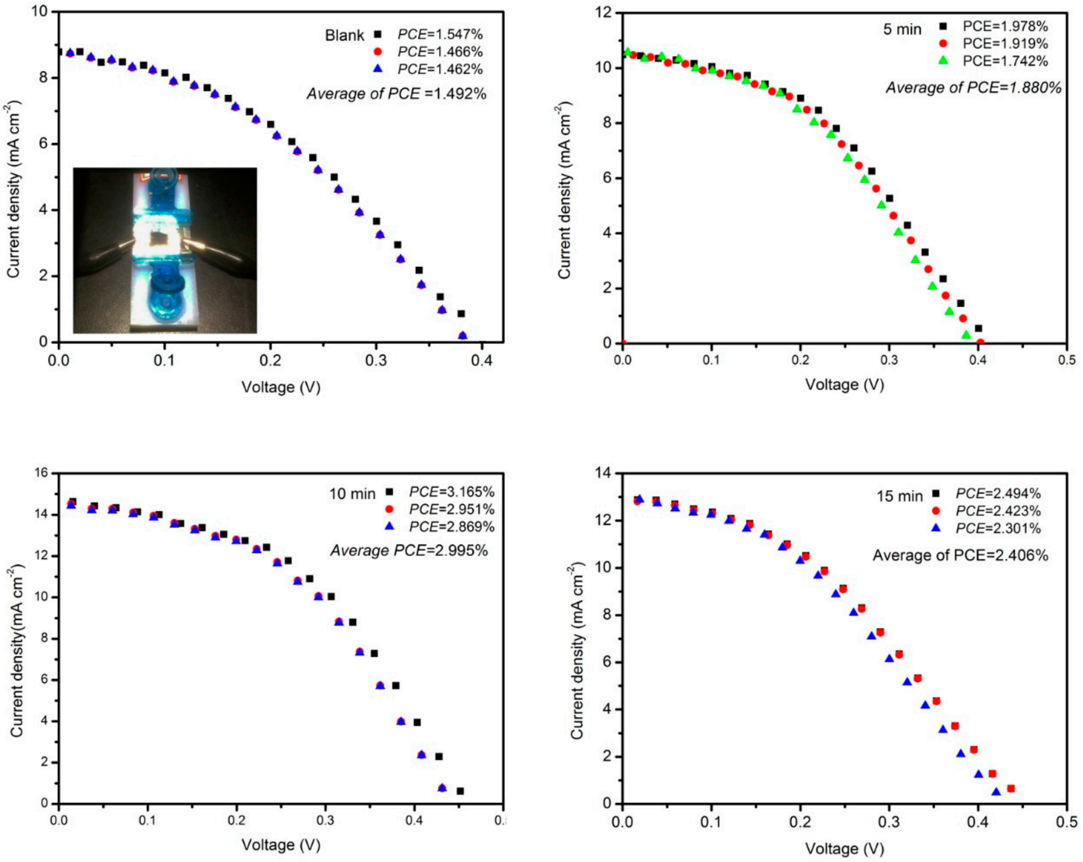

Figure S2. (**a–d**) *J-V* curves CdSe/CdS QDSSCs based on ZnO HMS passivated with TiO_2 by immersing in $TiCl_4$ aqueous solution for 0 min (blank), 5 min (5-min), 10 min (10-min) and 15 min (15-min), each solar cell have been repeatedly tested three times; the inset is a photograph of assembled solar cell in an open way for *J-V* test.

Table S1. Average photovoltaic performance and standard deviation extracted from *J-V* curves of QDSSCs based on ZnO HMS passivated with TiO_2 by immersing in $TiCl_4$ aqueous solution for 0 min (blank).

V_{oc} (V)	*Average* V_{oc} (V)	J_{sc} (mA cm^{-2})	*Average* J_{oc} (mA cm^{-2})	*FF*	*Average FF*	*PCE* (%)	*Average PCE* (%)	*Standard Deviation 0f PCE*
0.40		8.79		0.44		1.547		
0.39	0.393	8.72	8.750	0.43	0.433	1.462	1.492	0.048
0.39		8.74		0.43		1.466		

Table S2. Average photovoltaic performance and standard deviation extracted from *J-V* curves of QDSSCs based on ZnO HMS passivated with TiO_2 by immersing in $TiCl_4$ aqueous solution for 5 min.

V_{oc} (V)	*Average V_{oc}* (V)	J_{sc} (mA cm^{-2})	*Average J_{oc}* (mA cm^{-2})	*FF*	*Average FF*	*PCE* (%)	*Average PCE* (%)	*Standard Deviation 0f PCE*
0.41		10.49		0.46		1.978		
0.40	0.40	10.43	10.440	0.46	0.450	1.919	1.880	0.123
0.39		10.39		0.43		1.742		

Table S3. Average photovoltaic performance and standard deviation extracted from *J-V* curves of QDSSCs based on ZnO HMS passivated with TiO_2 by immersing in $TiCl_4$ aqueous solution for 10 min.

V_{oc} (V)	*Average V_{oc}* (V)	J_{sc} (mA cm^{-2})	*Average J_{oc}* (mA cm^{-2})	*FF*	*Average FF*	*PCE* (%)	*Average PCE* (%)	*Standard Deviation 0f PCE*
0.46		14.64		0.47		3.165		
0.44	0.447	14.58	14.570	0.46	0.460	2.951	2.995	0.483
0.44		14.49		0.45		2.869		

Table S4. Average photovoltaic performance and standard deviation extracted from *J-V* curves of QDSSCs based on ZnO HMS passivated with TiO_2 by immersing in $TiCl_4$ aqueous solution for 15 min.

V_{oc} (V)	*Average V_{oc}* (V)	J_{sc} (mA cm^{-2})	*Average J_{oc}* (mA cm^{-2})	*FF*	*Average FF*	*PCE* (%)	*Average PCE* (%)	*Standard Deviation 0f PCE*
0.45		12.89		0.43		2.494		
0.45	0.443	12.82	12.827	0.42	0.423	2.423	2.406	0.098
0.43		12.77		0.42		2.301		
